# Sudden cardiac death in anabolic androgenic steroids abuse: case report and literature review

**DOI:** 10.1080/20961790.2019.1595350

**Published:** 2019-08-19

**Authors:** Ana Isabel Hernández-Guerra, Javier Tapia, Luis Manuel Menéndez-Quintanal, Joaquín S. Lucena

**Affiliations:** a Histopathology Service, National Institute of Toxicology and Forensic Sciences (NITFS), Canary Islands Department, Tenerife, Spain;; b Forensic Pathology Service, Institute of Legal Medicine and Forensic Sciences (ILMFS), Las Palmas de Gran Canaria, Spain;; c Chemistry Service, NITFS, Canary Islands Department, Tenerife, Spain;; d Forensic Pathology Service, ILMFS, Seville, Spain

**Keywords:** Forensic sciences, forensic pathology, anabolic androgenic steroids (AAS), sudden cardiac death, autopsy, cardiac pathology, toxicology

## Abstract

Anabolic androgenic steroids (AAS) have several adverse effects on the cardiovascular system that may lead to a sudden cardiac death (SCD). We herein report a case involving a 24-year-old male, AAS abuser with intramuscular delivery in the 6 months before, who suffered a cardiorespiratory arrest at home’s bathtub when returning from New Year’s party. A forensic autopsy was performed according to the guidelines of the Association for European Cardiovascular Pathology (AECVP). The body showed hypertrophy of skeletal musculature, with low amount of subcutaneous fat and no signs of injury (body mass index, BMI: 26.8 kg/m^2^). On internal examination, there were multiorgan congestion, acute pulmonary edema, and cardiomegaly (420 g) with severe coronary atherosclerosis and superimposed acute occlusive thrombosis at the left main trunk and left anterior descendant. Areas of scarring were located at the intersection between the posterior wall and the posterior third of the septum (postero-septal). At histology, acute myocardial infarction at the anterior third of the septum and the anterior wall, and subacute myocardial infarction at apical septum and apical posterior wall were detected. Other findings were small intramyocardial vessel disease and myocytes hypertrophy. Chemicotoxicological analysis in blood showed ethanol ((0.90 ± 0.05) g/L), stanazolol (11.31 µg/L), nandrolone (2.05 µg/L) and testosterone (<1.00 µg/L). When confronted with a sudden death in a young athlete we must pay attention to the physical phenotype that may suggest AAS abuse and perform a detailed examination of the heart. Chemicotoxicological analysis is a key to establish the relationship between SCD and AAS abuse.

## Introduction

Anabolic androgenic steroids (AAS) are synthetic testosterone derivatives developed to increase strength and muscle mass (anabolic activity) and minimize androgenic activity [1,2].

AAS are used in clinical setting to counteract the several side effects on the treatment of several illnesses, such as osteoporosis, aplastic anemia, and hypogonadism among others, and cachexia-associated conditions as burns [3], HIV [4], renal and hepatic failure [5,6], and cancer [7].

The use of these agents has spread from athletes and body builders to adolescents and adults with the aim to enhance muscular development and athletic performance [8,9]. Illicit AAS use began to emerge in the 1980s in American population [10]. An estimated 2.9 to 4.0 million Americans have used supra-physiologic doses of illicit AAS [11]. About 1 million of individuals, most of them males, have developed AAS dependence, often leading to years of chronic AAS exposure [12,13]. The lifetime prevalence use in western countries typically ranges from 1% to 5% [14] with a global consumption of 6.4% in males and 1.6% in females [15].

Moreover, AAS users often associate other substances, the so-called “steroid accessory drugs”, such as insulin, diuretics, growth hormone (GH), ephedrine, gamma hydroxybutyric acid (GHB), erythropoietin, opiates, etc., with different purposes [8,16–18].

AAS have several adverse effects on the cardiovascular system: lipoprotein disorder, thrombosis, vasospasm, hypertension, cardiac hypertrophy, heart failure, arrhythmia, and sudden cardiac death (SCD) [19].

We present the case of a young male, AAS abuser with intramuscular delivery in the 6 months before, who died suddenly at home, describing the gross and microscopical findings at forensic autopsy, and toxicological results. The effects of AAS on the cardiovascular system are also reviewed.

## Case report

A 24-year-old male suffered a cardiorespiratory arrest at home's bathtub when returning from New Year's party. Cardiopulmonary resuscitation was unsuccessful and a forensic autopsy was ordered by the Magistrate on duty. According to the deceased's friends, he had taken AAS (stanozolol, testosterone, tamoxifen, mesterolone, and nandrolone) with intramuscular delivery in the previous 6 months (doses unknown). He had no family history of dyslipidaemia, premature atherosclerosis or cardiac events except for one episode of precordial pain some months before. There were no antecedents of illicit drugs consumption.

### Autopsy

**Figure 1.**
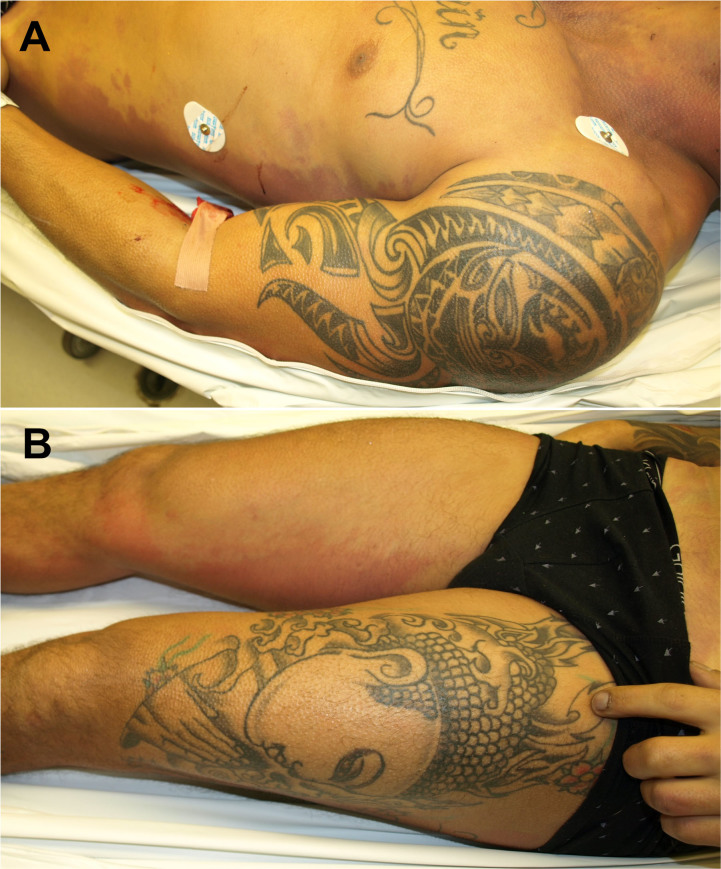
Hypertrophy of skeletal musculature and tattoos in shoulder, arm, thorax (A), and thigh (B).

A forensic autopsy was performed, with a postmortem delay of 12 h, according to the guidelines of the Association for European Cardiovascular Pathology (AECVP) [20]. The corpse showed hypertrophy of skeletal musculature, with low amount of subcutaneous fat and several tattoos in shoulders, arms, thighs, and thorax (Figure 1). The height was 178 cm and the weight 85 kg (body mass index, BMI: 26.8 kg/m^2^). On internal examination, there were multiorgan congestion and acute pulmonary edema. Mechanical trauma or asphyxia was ruled out.

**Figure 2.**
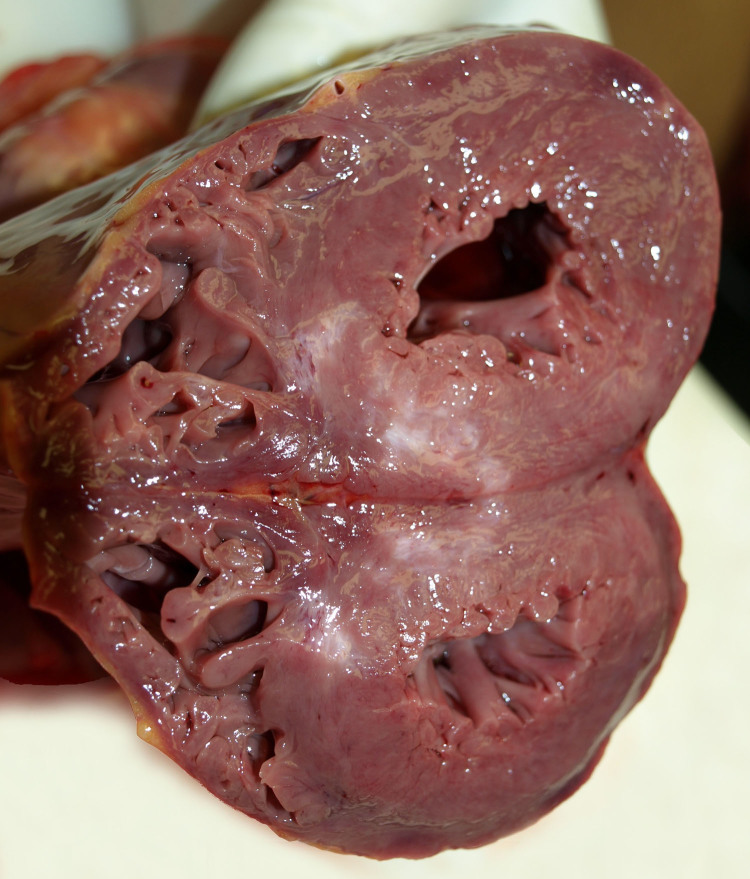
Macroscopic examination of the heart: areas of scarring located at the intersection between the posterior wall and the posterior third of the septum (postero-septal).

**Figure 3.**
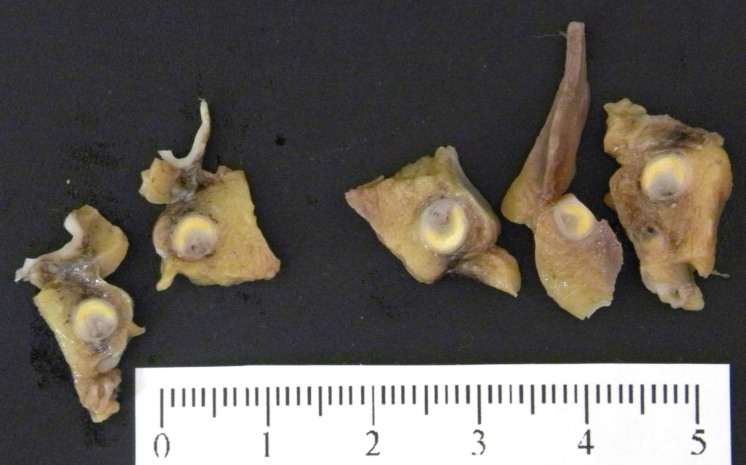
Macroscopic examination of the coronary arteries: severe atherosclerosis with acute occlusive thrombosis at the left main trunk and left anterior descendant.

**Figure 4.**
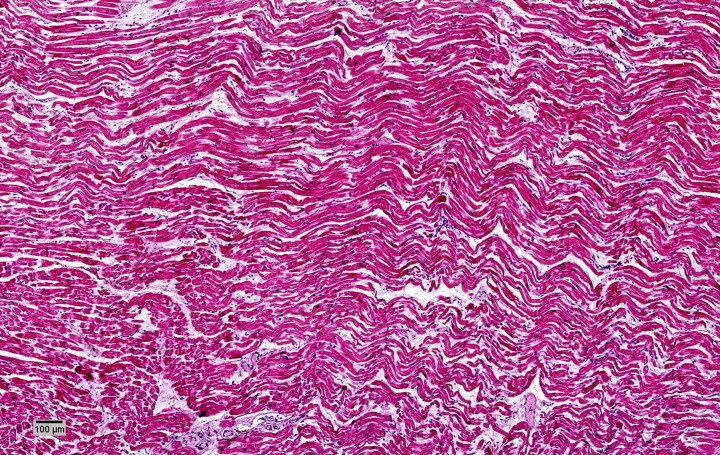
Early acute myocardial infarction at the anterior third of the septum and left ventricle anterior wall: wave fibers with elongation and narrowing as an early sign of acute ischemic damage and mild edema (HE, ×10).

**Figure 5.**
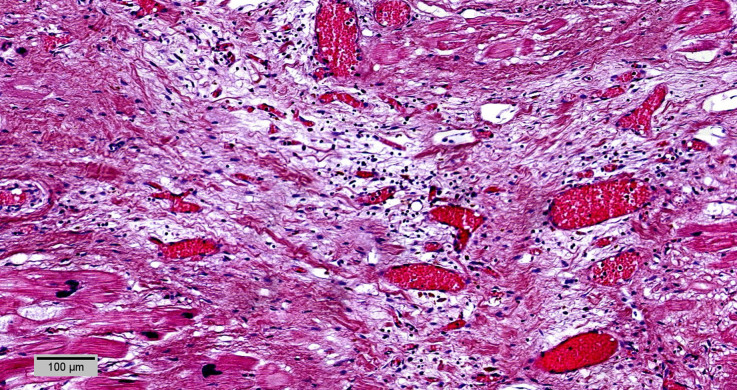
Subacute myocardial infarction at the left ventricular free wall and septum: loose connective tissue with capillaries and inflammatory infiltrate (HE, ×10).

**Figure 6.**
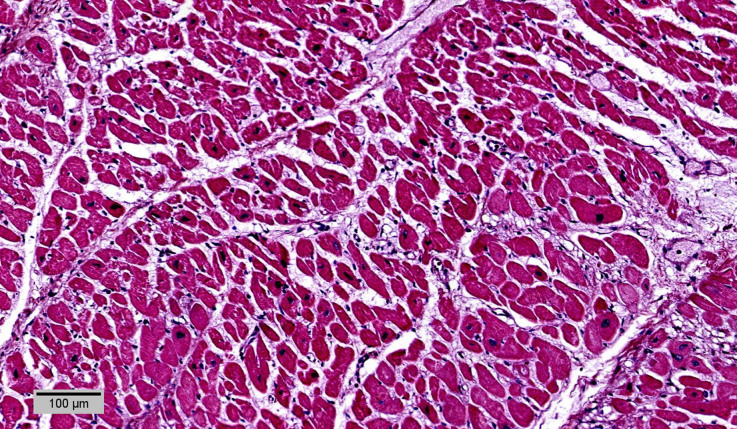
Myocytes hypertrophy with dysmetric and dysmorphic nuclei (HE, ×10).

**Figure 7.**
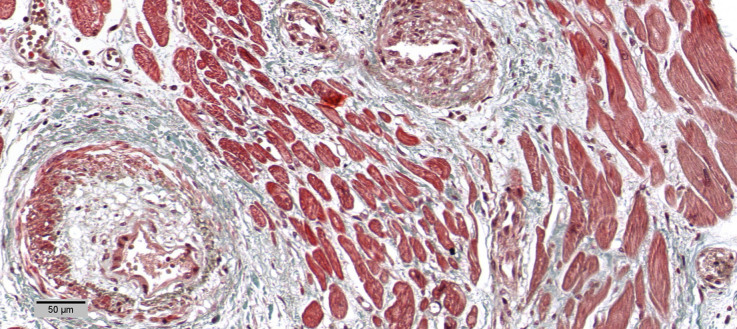
Small intramyocardial vessels disease with media hypertrophy (Masson Trichrome 20×).

**Figure 8.**
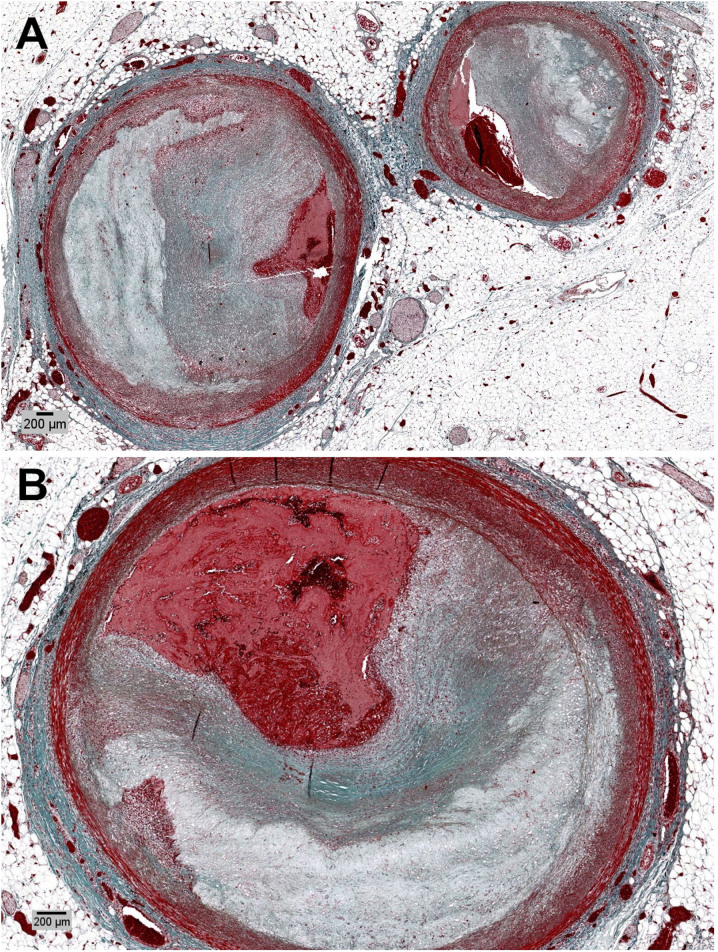
Severe atherosclerosis with acute occlusive thrombosis at the (A) left main trunk (Masson Trichrome, ×2), and (B) left anterior descendant (Masson Trichrome, ×4).

On gross examination the heart showed cardiomegaly (420 g) being the expected heart weight in relation to body weight of 358 g (range 271–473 g) according to Kitzman et al. [21]. The ventricular thickness was within the upper normal ranges (left ventricular free wall 15 mm, ventricular septum 15 mm, right ventricular free wall 5 mm) (Figure 2). Coronary arteries, with a right dominance, had a normal origin and course, showing severe atherosclerosis (>75% stenosis) with acute superimposed occlusive thrombosis at the left main trunk and left anterior descendant (LAD) (Figure 3). The right and circumflex coronary arteries did not show alterations. Areas of scarring were located at the intersection between the posterior wall and the posterior third of the septum (postero-septal) (Figure 2). Atrioventricular and sigmoid valves were normal. For histology, samples of ventricular myocardium (*n* = 14), coronary artery, brain, lung, liver and kidney were taken, fixed in formaldehyde solution 4%, buffered, paraffin-embedded sectioned at 3–6 µm thickness and stained with hematoxylin and eosin (HE) and Masson trichrome (heart). Acute myocardial infarction at the anterior third of the septum and the left ventricle (LV) anterior wall (Figure 4), subacute myocardial infarction at apical septum and apical posterior LV wall (Figure 5), myocytes hypertrophy (Figure 6) and small intramyocardial vessel disease (Figure 7) were detected. Furthermore, the macroscopic findings described in the coronary arteries were confirmed (Figure 8). The rest of organs showed congestion and acute pulmonary edema.

### Toxicological analysis

The peripheral blood was obtained from femoral vein (adding potassium oxalate and sodium fluoride as preservatives) and stored refrigerated at 4 °C until the analysis. Blood and vitreous humor were used for routine ethanol and other volatiles screening by headspace gas chromatography with flame-ionization detector (HS-GC-FID) (Varian 3800CP; Varian, Inc., Walnut Creek, CA, USA) coupled with combipal autosampler (CTC Analytics, Zwingen, Switzerland). Blood was screened by cloned-enzyme donor immunoassay (CEDIA) (Indiko^®^; Thermo Fisher Scientific, Waltham, MA, USA) for the presence of cocaine, amphetamines, methadone, barbiturates, opiates and cannabis. Then, independently of the result obtained in the immunoassay, blood and vitreous humor were submitted to a broad toxicological analysis using gas chromatography (Agilent 6890N; Agilent Technologies, Santa Clara, CA, USA), with mass spectrometric (MS) detection (Agilent 5973; Agilent Technologies) in combination with high-performance liquid chromatography (HPLC) (Agilent Infinity 1260 G1311B, Agilent Technologies) with a diode-array detector (DAD) (Agilent Infinity 1260 G4212B; Agilent Technologies). Analysis of anabolic steroids in whole blood was performed using a method previously developed by Fabresse et al. [22] using liquid chromatography (Vanquish UHPLC; Thermo Scientific, Waltham, MA, USA) coupled to high-resolution mass spectrometry (LC-HRMS) (Orbitrap Q-Exactive-Focus; Thermo Scientific).

#### Alcohol analysis procedure and results

Ethanol was quantified by HS-GC-FID (Varian 3800CP) using the official method of the National Institute of Toxicology and Forensic Sciences (NITFS). The column used was a DBWAX (30 m × 0.25 mm × 0.25 µm, Agilent J&W GC Columns; Agilent Technologies). To 200 µL of each specimen was added another 200 mL of saturated saline solution containing 1-propanol as internal standard. After stirring and waiting 15 min to reach the equilibrium 500 µL of headspace was injected into the chromatograph. Ethanol was positive in blood ((0.90 ± 0.05) g/L) and vitreous humour (0.84 g/L). 

#### Preparation of blood and vitreous humor for routine toxicological analysis by GC-MS and HPLC-DAD and results

Blood and vitreous humor were submitted to systematic toxicological analysis by solid phase extraction (SPE). The pH of each sample 2.5 mL of blood and 2.5 mL of humor vitreous was adjusted to 6. The mixture was poured into Bond-Elut Certify™ columns (Agilent Technologies), and gently sucked through. Finally, the analytes were eluted with 3 mL of a freshly prepared mixture of chloroform:acetone (*V*_chloroform_:*V*_acetone_ = 50:50) and then with a second mixture of dichloromethane:isopropanol:ammonia (*V*_dichloromethane_:*V*_isopropanol_:*V*_ammonia_ = 78:14:8). The elutes were collected and evaporated to dryness under a gentle nitrogen stream. The residue was reconstituted with 100 µL of methanol and transferred to a chromatographic vial.

Aliquots (2 µL) of the extracts were injected into a GC–MS system mentioned above. In this case, a VF-1 ms capillary column (methylsilicone gum, 30 m × 0.25 mm × 0.25 µm; Hewlett Packard, San Jose, CA, USA) was used. In parallel, 4 µL of each extract were injected into the HPLC-DAD system mentioned above. In this case, a gradient created by a mobile phase composed of aqueous phosphate 10 mmol/L buffer pH 3.6 and acetonitrile which allows to obtain the complete separation of the analytes on a C18 reversed phase column (250 m × 4.6 mm × 5 µm; Kinetex, Torrance, CA, USA).

No toxic substances were found in this routinely analysis.

#### Analysis of anabolic steroids in whole blood

One milliliter of methanol and 2 mL of sodium hydroxide 0.1 mol/L were added to 2 mL of whole blood, then the mixture after stirring was extracted twice with 2 mL of heptane. The sample was mixed for 15 min and then centrifuged at 3 500 rpm for 10 min. The upper organic layer was decanted into another tube and evaporated to dryness under a nitrogen steam. The sample was reconstituted with 100 µL of methanol containing 0.1% formic acid, vortex mixed for 10 s, and transferred into injection vials for analysis.

A six-point calibration curve was used over the range 1.0 to 100.0 µg/L for blood. The calibration levels were prepared spiking 2 mL of physiological serum with a standard solution containing stanozolol, nandrolone, tamoxifen, testosterone, and mesterolone and submitted to the same procedure as whole blood.

Chromatography was performed on a Thermo Vanquish UHPLC system and separation was carried out on a C18 Thermo column (100 m × 2.1 mm × 1.9 µm; Thermo Scientific) maintained at 30 °C. The mobile phase was composed of solvents A (aqueous 0.05% formic acid) and B (acetonitrile + 0.1% formic acid). The column was maintained at 35 °C and eluted with a gradient of 10% B (0–1.0 min), 10%–100% B (1.0–5.0 min), hold at 100% B (5.0–6.0 min); the column was finally set to 10% B (6.0–6.5 min) and hold at 10% B (6.5–9.0 min). The total runtime was 9 min at a flow rate of 0.40 mL/min. Compounds were detected using an Orbitrap mass spectrometer (Q-Exactive Focus; Thermo Scientific) equipped with a heated electrospray ionization source (HESI) operating in positive ionization mode (Sheath gas flow rate 60, Aux gas flow rate 5, Spray Voltaje 3.50, Capillary Temp 380 °C, S-lens 300, Aux gas heat Temp 300 °C). Data were acquired in PRM mode over a mass range of 100–400 *m*/*z* at the resolving power 35.000 with an isolation window of 3.0 *m*/*z*. Chromatographic data acquisition and quantification were performed using TraceFinder Forensic v4.1 software (Thermo Scientific).

Protonated molecular ions (M + H^+^) of steroids were identified with exact mass and retention time (±0.2 min). Mass error was <5 ppm for all analytes. No interferences were observed at the retention times. The method showed a good linearity in the range previously described in physiological serum. The lower limit of quantification for all compounds was 100 µg/L.

The results from the analysis of those anabolic steroids in blood are summarized in Table 1. Tamoxifen and mesterolone were not found and testosterone was below quantification level.

**Table 1.** Results of anabolic steroids determination.

Analyte	Blood (µg/L)
Stanozolol	11.31
Testosterone	<1.00^a^
Tamoxifen	ND
Mesterolone	ND
Nandrolone	2.05

a Below the quantification level.

ND, no detection.

## Discussion

The cause of death in this young male was myocardial infarction with severe coronary atherosclerosis and acute occlusive thrombosis affecting left main trunk and LAD (single vessel disease) secondary to AAS consumption. Personal antecedents and chemicotoxicological analyses excluded the presence of any other drugs of abuse. He had no family history of dyslipidaemia, premature atherosclerosis, or cardiac events.

Cardiovascular effects of AAS described in case reports are mainly related with acute myocardial infarction due to premature atherosclerosis. Myocardial infarction without significant coronary atherosclerotic disease has also been reported [9,23–26]. Other adverse cardiovascular effects such as left ventricular hypertrophy, impaired left ventricular function, arterial thrombosis, and pulmonary embolism have been described [9,16,23,27–31]. The most typical myocardial abnormality in AAS abusers is left ventricular hypertrophy, associated with fibrosis and myocytolysis [8,9].

Similar lesions related with premature coronary atherosclerosis, affecting mainly the LAD, and acute myocardial infarction has been described in cases of SCD in young people (<35 years old) due to cocaine [32] or amphetamine consumption [33].

Acute non-fatal myocardial infarction was first reported in 1988 [30] and fatal myocardial infarction in 1990 [32]. Up until today, 19 fatal cases (89.5% males), age ranged 18–37 years, have been reported in medical literature [9,23,27,28,34–39], but with only one case of occlusive thrombus in left coronary artery [35].

Melchert and Welder [40] suggested that there are at least four hypothetical models of ASS-induced adverse cardiovascular effects: atherogenic, thrombosis, vasospasm, and direct myocardial injury. The changes in lipid metabolism and lipoprotein levels increase the risk of atherosclerosis; polycythemia and enhanced platelet aggregation increase the risk of thrombus formation, and arterial vasospasm increases the risk of ischemia and the occurrence of infarction [6,19,41]. Adverse effects of AAS on the diastolic and systolic function are probably due also to direct toxicity on myocardial structure (apoptosis) with increased collagen deposition, fibrosis, altered microcirculation with intimal hyperplasia of the intramural coronary arteries resulting in chronic ischemic damage [9]. Vascular endothelial cells may be directly affected by AAS, which may result in vasospasm [40]. All of these mechanisms associate AAS use with a high risk of SCD.

Some authors have proposed that at physiological doses of testosterone, androgen receptors are saturated and the anabolic effects of supra-physiological doses of AAS occur via interaction of these synthetic androgens with glucocorticoid receptors [6,42,43]. AAS have a low affinity for glucocorticoid receptors, but, at high concentrations, they can inhibit the binding of glucocorticoids and block their catabolic effects [6,44,45].

Cardiovascular responses to AAS are due to specific myocardial receptors, which have transcriptional regulatory functions. The cardiac hypertrophy induced by AAS appears to be generated by a direct action on cardiac androgen receptors, whose effects are directly proportional to the dose, time and duration of drug administration [9,46–48].

The sympathetic nervous system involved in the neurological control of the cardiovascular system may be influenced by AAS when combined with exercise and confer an increased risk of life-threatening arrhythmias [14,49].

According to Achar et al. [50], AAS are often consumed concomitantly with GH, erythropoietin, and other agents. This is important because GH may lead to cardiomyopathy, abnormal lipoprotein profiles [51,52], and left ventricular hypertrophy [53]. Erythropoietin abuse is linked to hypertension and increased risk for thromboembolic events [54]. These effects may be difficult to separate from the results of AAS abuse alone and motivate the further need for more rigorous clinical and forensic screening.

We are in agreement with other authors in the consideration that nowadays AAS abuse is a public health issue because of self-administration that is particularly widespread among no-athletes at fitness centers with aesthetics goals [6,28,55,56].

## Conclusion

When confronted with a sudden death in a young athlete we must pay attention to the physical phenotype such as muscular hypertrophy, striae in pectoral or biceps muscle, gynecomastia, testicular atrophy, and acne that may suggest AAS abuse and perform a detailed examination of the heart. Chemicotoxicological analysis is key to establish the relationship between SCD and AAS abuse.
